# The fracture resistance of pulpotomized primary molars restored with zirconia crowns, lithium disilicate or resin based ceramic endocrowns

**DOI:** 10.1186/s12903-024-04332-4

**Published:** 2024-05-20

**Authors:** Amany Abd Elhady Muhammad Ali, Asmaa Ali Emam Abo-ELsoud, Yousra Samir Helmy

**Affiliations:** Department of Pediatric Dentistry, Preventive Dentistry and Dental Public Health, Faculty of Dentistry, Suez Canal University, Ismailia, Egypt

**Keywords:** Primary, Molars, Endocrown, E-Max, Brilliant crios

## Abstract

**Background:**

Endocrown in pediatric dentistry was rooted in the fundamental principles of preserving healthy dental tissues, leveraging contemporary adhesive methodologies.

**Aim:**

This research aimed on assessing and comparing the fracture resistance of pulpotomized primary molars when rehabilitated with zirconia crowns and two distinct types of endocrowns, namely E-Max and Brilliant Crios.

**Methods:**

The study involved thirty, anonymized, freshly extracted second primary molars that underwent pulpotomy. These teeth were then evenly divided into three groups, each consisting of ten specimens: the zirconia crown, the E-Max endocrown, and the Brilliant Crios endocrown groups. Post-pulpotomy, the teeth were prepared for their respective restorations. Subsequent to this preparation, the zirconia crowns, E-Max endocrowns, and Brilliant Crios endocrowns were secured. To evaluate the fracture resistance using a computer-controlled testing machine (Instron), a progressively increasing load was applied to each group until fracture occurred. The gathered data were then analyzed for outliers and subjected to normality testing using the Shapiro-Wilk and/or Kolmogorov-Smirnov tests, with a significance threshold set at 0.05.

**Results:**

There was no statistically significant difference in fracture resistance of pulpotomized primary molars among lithium disilicate (E-Max) group (mean=1367.59N), Brilliant Crios group (mean=1349.73N) and zirconia group (mean=1240.82N).

**Conclusion:**

Endocrowns can be considered a promising restoration for pulpotomized primary molars.

## Background

Deciduous teeth are crucial for mastication, speech, aesthetic appeal, preserving space for the eruption of permanent teeth, and stimulating maxillary and mandibular development [1]. In cases of primary molars affected by extensive carious lesions, pulpotomy is often necessitated. Such pulpotomized molars share similarities with teeth that have undergone endodontic treatment, exhibiting increased fragility primarily due to the reduction in structural integrity after pulp therapy. Hence, when choosing materials for restoring these devitalized teeth, it is imperative to select those that not only reconstruct the lost dental structure but also enhance mechanical and functional integrity, aesthetic quality, and coronal sealing [2, 3].

For decades, stainless steel crowns (SSCs) have been the standard restoration choice post-pulpotomy in primary molars. SSCs provide effective protection for weakened dental structures and help in preventing marginal leakage, thus ensuring an adequate coronal seal. However, their aesthetic appeal is limited, and they are not suitable for patients with metal allergies [4, 5].

The increasing demand for restorations, that are both functional and visually appealing, influenced by the preferences of patients and their guardians. This has provoked the creation of aesthetic full-coverage restorations, such as Zirconia Crowns. Constructed from polycrystalline ceramic that lacks a glass component, zirconia crowns exhibit notable characteristics including high compressive strength, corrosion and fracture resistance, biocompatibility, and long-lasting durability, all while offering an aesthetically attractive finish [6]. However, zirconia crowns require more extensive tooth preparation, leading to longer treatment times, and they tend to cause significant wear on the enamel of opposing teeth [7].

First conceptualized by Bindl and Mörmann in 1999, endocrown restorations leverage the pulp chamber for central retention while employing adhesive resin cement for microretention. These restorations are optimally suited for molars exhibiting specific conditions, including dilaceration, shortness, obliteration, or fragility of roots, substantial loss of coronal dental tissue, and limited oral opening. These are common challenges faced in pediatric dentistry, particularly in the treatment of pulpotomized primary molars [8, 9]. The initial endocrown restorations were crafted from porcelain blocks using CAD/CAM technology in the CEREC system. Subsequently, resin materials, known for their stress-absorbing properties, were also employed in endocrown fabrication [10].

Advancements in dental adhesive technology and materials have paved the way for indirect composite or ceramic restorations created using CAD/CAM systems in inlay ovens, which are particularly beneficial in pediatric dentistry due to their reduced treatment time, thereby enhancing patient cooperation [4, 11]. Lithium disilicate-based ceramics (e.g., E-max) are noted for their high mechanical strength, excellent bonding properties, and superior aesthetics, given that the dimensions of the ceramic used in endocrowns are more substantial than those in standard ceramic crowns [12, 13]. Resin matrix ceramics like Brilliant Crios, with their stress-absorbing characteristics, are advantageous for patients with weaker periodontium. Moreover, indirect resin-based endocrowns are less abrasive to opposing natural teeth compared to ceramic ones and can be intraorally repaired with composites [14].

While endocrowns have gained popularity in adult dentistry, as there is plenty of reviews discussing the application of endocrowns in restoring permanent endodontically treated teeth, their application in restoring pulpotomized primary molars has not been broadly investigated. By focusing specifically on the pediatric population, this study will contribute in filling the knowledge gap in the current literature by investigating the efficacy of endocrowns in pediatric dentistry. The findings of this research have the potential to provide valuable insights into the selection of the most suitable restorative option for pulpotomized primary molars, while offering valuable insights to clinicians, and researchers. As fracture resistance of pulpotomized primary molars is a critical factor to be considered during rehabilitation. The restoration of these teeth plays a significant role in maintaining their structural integrity and functionality. Therefore, the aim of this study is to assess and compare the fracture resistance of pulpotomized primary molars rehabilitated with zirconia crowns and two distinct types of endocrowns, namely E-Max and Brilliant Crios.

## Methods

The study's procedures received approval from the Research Ethics Committee (REC) of the Faculty of Dentistry, Suez Canal University, under the authorization number 3119/2021, in accordance with the Helsinki Declaration of the World Medical Association (2008 Version). This study included thirty, unidentifiable, primary, second molars, that were extracted in the Pediatric Dentistry and Oral and Maxillofacial Surgery Departments, Suez Canal University, for reasons unrelated to this research, such as natural exfoliation or orthodontic interventions. All the patients under 16 years old who presented to these above-mentioned departments had their parents or legal guardians sign informed consent forms to use their removed teeth for study purposes. The sample size was determined using G*Power software version 3.1.9.2 [15], based on an effect size of 0.80, an alpha (α) level of 0.05, and a Beta (β) level of 0.20, corresponding to a power of 80%. Consequently, the calculated minimum number of samples required was thirty.

### Sample selection

Extracted mandibular second primary molars were selected with sufficient tooth structure (sound teeth or simple class I), with at least one-third of the root still present. The selected teeth had a similarity in buccolingual and mesiodistal dimensions allowing for ±1mm difference of tooth three dimensions, using a digital caliper. Molars with macroscopic defects (cracks, hypoplasia, hypomineralization) were excluded with the aid of operating microscope [2].

#### Sample storage and disinfection

The chosen teeth were sterilized using 0.1% thymol solution and then preserved in distilled water at ambient temperature. This storage was maintained for no longer than one month prior to their utilization in the experiment [16].

#### Sample Mounting

The selected teeth were mounted using a specially designed Teflon cubic split mold, the mold was filled with chemically cured acrylic resin. The tooth was inserted vertically in the center of the mold. The root of the tooth was encased in acrylic resin (Technovit 4000, Heraeus Kulzer, Hanau, Germany), ensuring that the cemento-enamel junction was positioned 2 mm above and aligned parallel to the surface of the acrylic resin [17].

#### Pulpotomy procedure

**Fig. 1:**
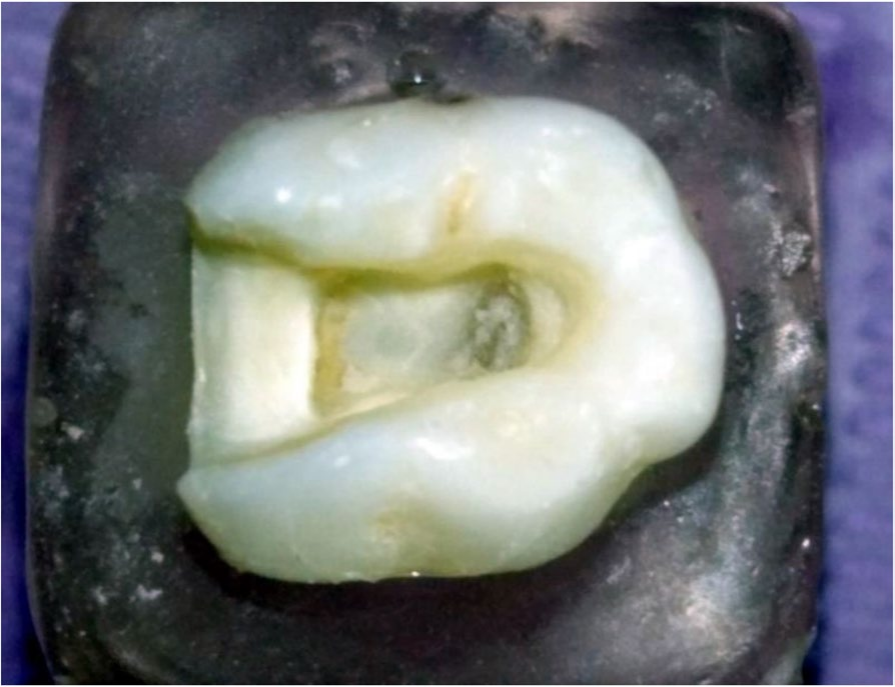
Pulpotomized second primary molar

All mounted molars will be pulpotomized as per the guidelines outlined in reference [18], following these steps:

1. Molars, with dental caries, were excavated using a round steel dental bur (number 2, featuring a 1mm head diameter).
2. The access cavity walls were expanded to facilitate the de-roofing of pulp chamber.
3. The preparation of a Class II cavity was executed using an inverted cone bur of carbide material (number 2) and fissure carbide burs (number 2). For uniformity in the procedure, the gingival wall was constructed 1 mm above the cementoenamel junction and was given a buccolingual dimension of 3 mm. The walls of the pulp chamber were contoured to have an angular divergence ranging from 6° to 8°, achieved using a tapered cylindrical diamond bur (number 837-012) (Fig. 1).
4. Subsequently, the pulp chamber was meticulously cleaned to remove any remaining pulpal tissues. This was followed by a thorough rinsing with water, and then drying using air flow.
5. A dense mixture of zinc-oxide and eugenol (ZOE) paste (I dental, AUB, Lithuania) was applied into the cavity with a thickness of 2mm. Any extra paste was then, carefully removed from the cavity walls.
6. A 2mm thick layer of self-curing glass ionomer cement (RIVA self-cure, B dent, Australia) was, then, uniformly applied [19].

### Sample randomization and grouping

The sample of thirty teeth was numerically labeled from 1 to 30. These samples were then categorized into three distinct groups, each consisting of ten teeth, using the randomization service provided by www.randomizer.org. The division was based on the restoration type and materials employed, detailed as follows:

- Zirconia Crown Group: This group, serving as the control, consisted of ten pulpotomized primary molars. These teeth were restored using pre-fabricated zirconia crowns supplied by Nu smile, USA.
- E-max Group: In this group, ten pulpotomized primary molars were restored using endocrowns made of Lithium disilicate (the E-max brand from Ivoclar Vivadent, AG).
- Brilliant Crios (composite) Group: Ten pulpotomized primary molars were restored with resin matrix ceramics (brilliant crios, Swizerland) endocrowns.

### Restoration of the pulpotomized molars

- Zirconia Crown Group Preparation Methodology:

The preparation for this group was meticulously carried out using a specialized standardization device, AF30, from Switzerland, and employed coolant during the process as detailed below [20]:

- Occlusal Reduction: An occlusal preparation involving a reduction of 1.5-2 mm was completed.
- Buccal and Lingual Reduction: The buccal and lingual surfaces were reduced, ensuring a minimum tooth structure removal of 0.5–1.0 mm.
- Proximal Slicing: Proximal mesial and distal slicing was performed, culminating in a tapered edge, akin to a knife or feather edge, at the gingival margin. This tapering varied in thickness from 0.5 to 1.25 mm.
- Circumferential Reduction: The expected edge resulting from the circumferential reduction was finished into a featheredge. This was done to facilitate the passive fitting of the zirconia crown.

- E-max and Brilliant Crios endocrown group preparations:

The standardization of endocrown preparations for the E-max and Brilliant Crios groups was executed using a customized milling machine (AF30, Switzerland), accompanied by the use of a coolant, in accordance with the protocol detailed in reference [2], as outlined below:

A) **Occlusal reduction/clearance:**

Cuts with a depth of 1.5mm were precisely created on the occlusal surface. The vertical dimension of these cuts was meticulously maintained at 1.5 mm, and the horizontal plane was variably modified to achieve thorough clearance of the surface. This process was critical in establishing the location of the cervical margin, commonly known as the "cervical sidewalk" or the "butt joint" finish line.

B) **Axial wall preparation:**

The axial walls were consistently prepared to create a 7-degree angle of divergence, utilizing the identical standardization equipment that features a specialized milling machine fitted with a tapered stone.

C) **Smoothing and rounding internal margins:**

**Fig. 2:**
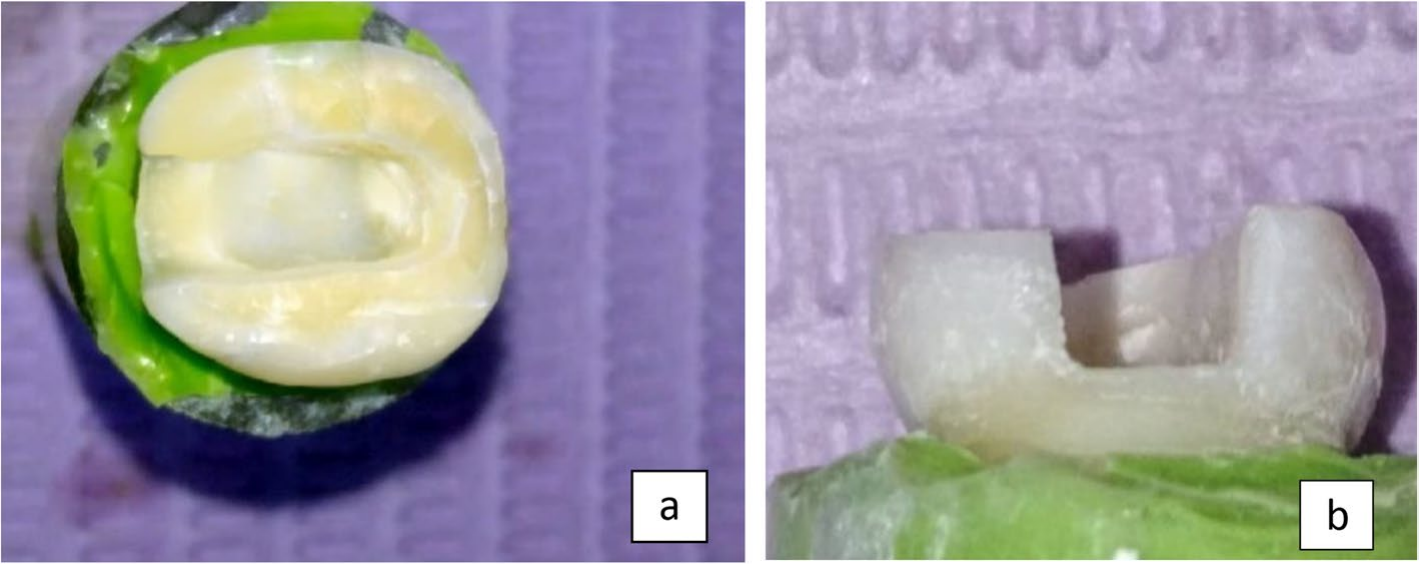
**a** & **b** Endocrown molar preparation

The procedure began by employing the identical diamond tip to initially smooth and round off the internal angles at the margins. This was followed by a finishing phase where an abrasive rubber tip was used to polish the internal angles, resulting in a preparation that is both polished and smoothed, as shown in (Fig. 2a & b).

**Fig. 3:**
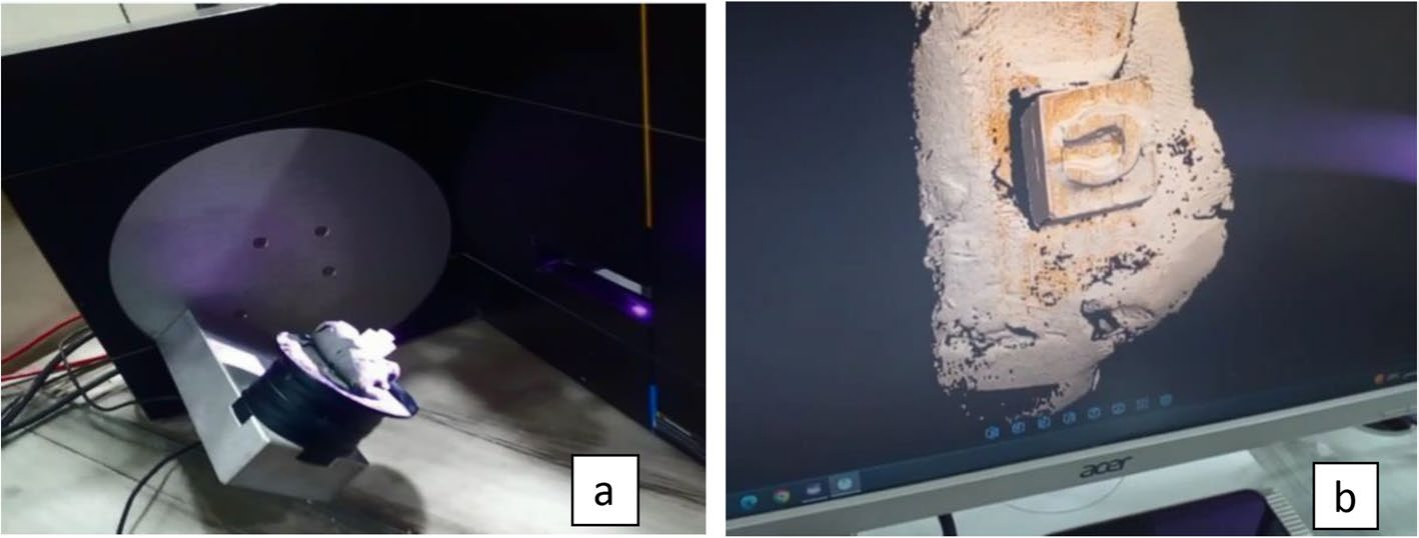
**a** & **b** Scanning step

D) **Laboratory Phase** [21]:

During the laboratory phase, the Exocad dental system (Exocad GmbH, Version 3.0, Darmstadt, Germany) was utilized for the fabrication of the endocrowns. The teeth prepared for endocrown placement underwent a three-dimensional imaging process. Initially, a Telescan light-reflecting powder from DOF, Korea, was applied to each prepared tooth. This preparation facilitated the acquisition of an optical impression of the sample. Subsequently, the Identica Blue scanner (3D scanner DOF, Korea) was employed for scanning the samples, and the captured images were saved. An automated margin detection tool was then used to precisely identify the margins of the preparation, as illustrated in (Fig. 3a & b).

**Fig. 4:**
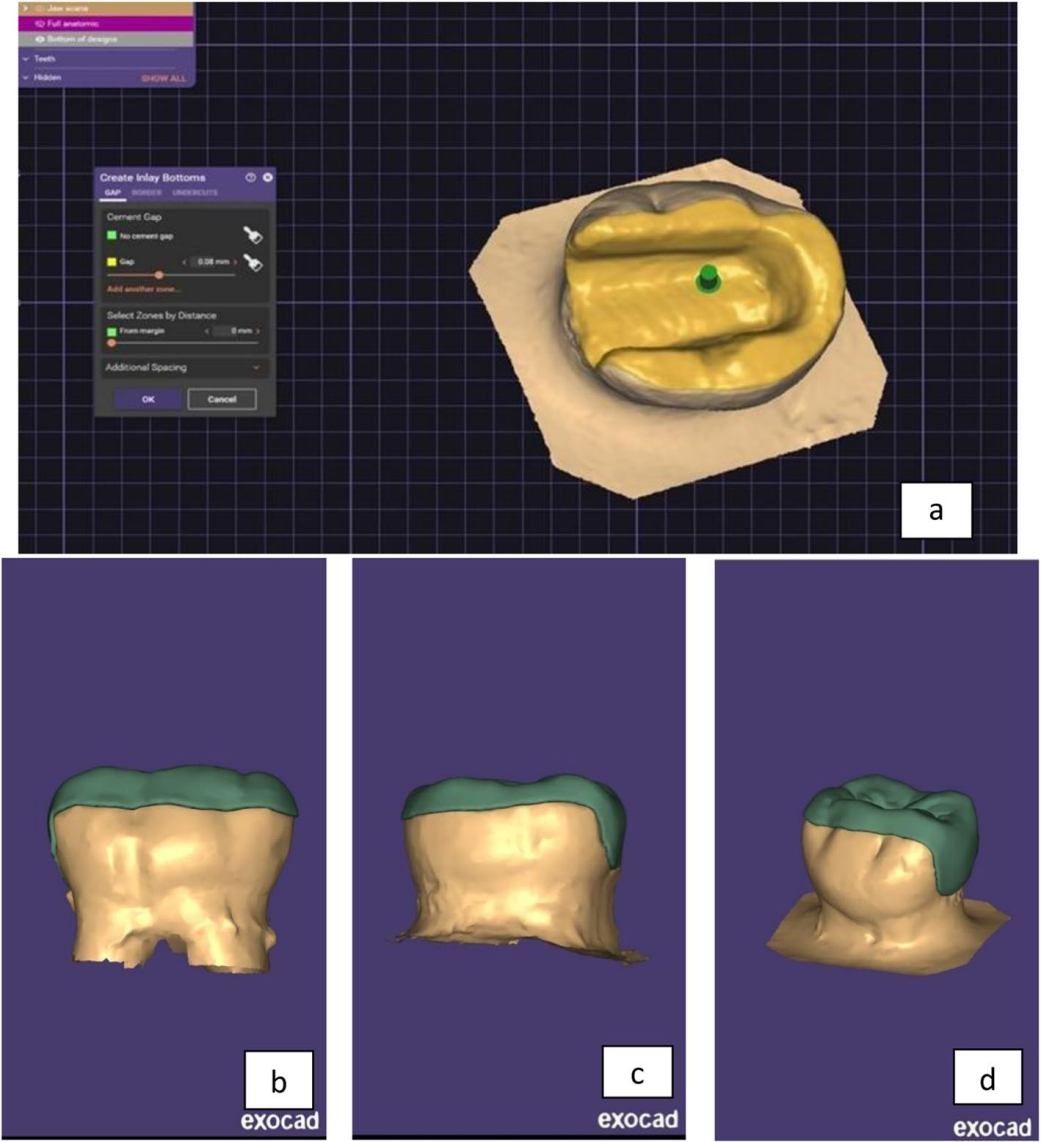
**a**, **b**, **c** & **d** Designing of endocrown by exocad

**Fig. 5:**
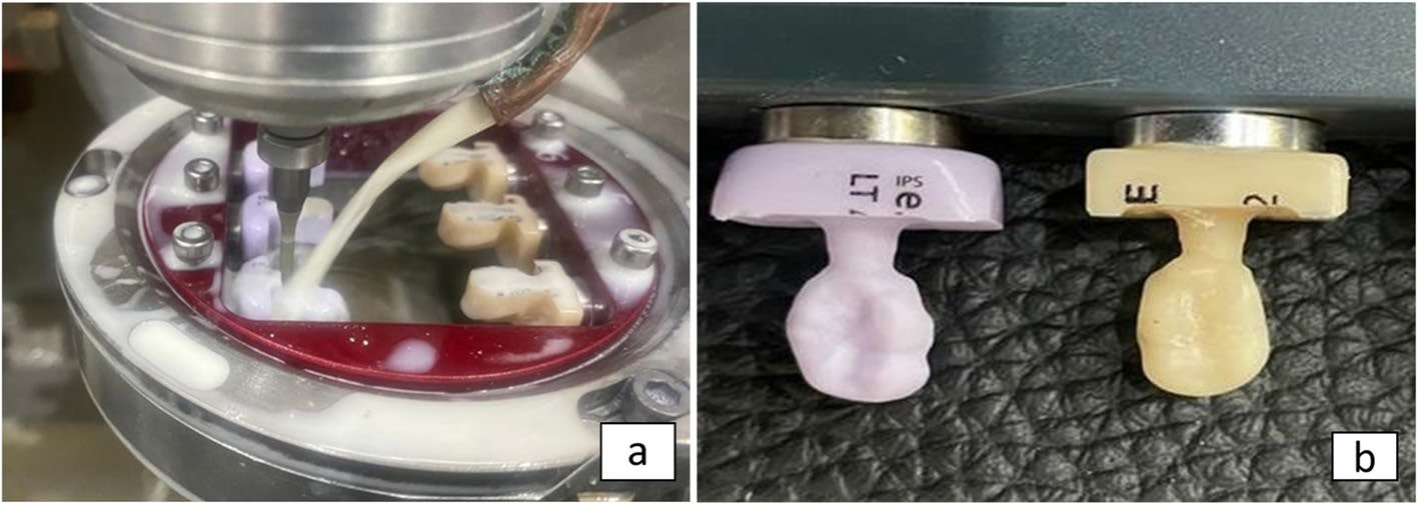
**a** & **b** Milling step by CAD/CAM

- Standardization of restoration design for endocrown specimens: The design parameters for the endocrown specimens were uniformly set using the Exocad dental system, supported by the biogeneric copy feature. This ensured consistency across all endocrown designs.
- 3D virtual endocrown construction: The 3D imaging of the prepared tooth, captured during the scanning phase, was utilized to create a virtual model of the endocrown. This model incorporated a precise cement gap of 0.08mm, as shown in (Fig. 4a, b, c & d).
- Initiation of the Milling Process: For the milling of the endocrowns, blocks designated for this purpose were affixed to the spindle arm of the CAD/CAM milling machine (Arum, Germany).
- Automated Milling with Water Irrigation: The milling operation was fully automated and included the application of abundant water irrigation from multiple directions to ensure optimal processing, as shown in (Fig. 5a & b).

### Glaze Step for E-Max endocrowns

In their initial state post-milling, the E-Max endocrowns exhibited a bluish-gray hue, indicative of their pre-crystallized form. The crystallization and glaze firing of these endocrowns were carried out using a Programat P100 furnace from Ivoclar AG, Austria.

**Fig. 6:**
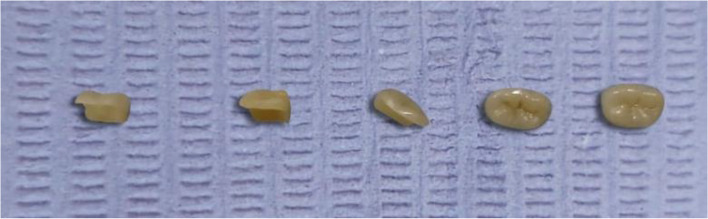
Finally glazed E-max endocrown

In the firing phase, E-Max endocrowns were stabilized with an object-fixing substance and positioned on their specific firing trays as per the manufacturer's instructions. The firing sequence initiated at 403°C and escalated at a pace of 90°C per minute, peaking at 840°C. This temperature was sustained for 7 minutes, finalizing the development of the E-Max endocrowns. This process is illustrated in Fig. 6.

### Preparation of internal surface for endocrown bonding [22]

#### For E-max group

In the E-max group, a 9.5% HF acid solution (9.5% acid Bisco, USA) specifically designed for porcelain etching was applied to the internal surfaces of the endocrowns for a duration of 20 seconds. Followed by, a comprehensive rinse lasting 60 seconds, and the surfaces were then air-dried. Afterwards, Porcelain Silane (Pentron, USA) was applied with a brush and allowed to dry for a period of 60 seconds.

#### For brilliant crios group

**Fig. 7:**
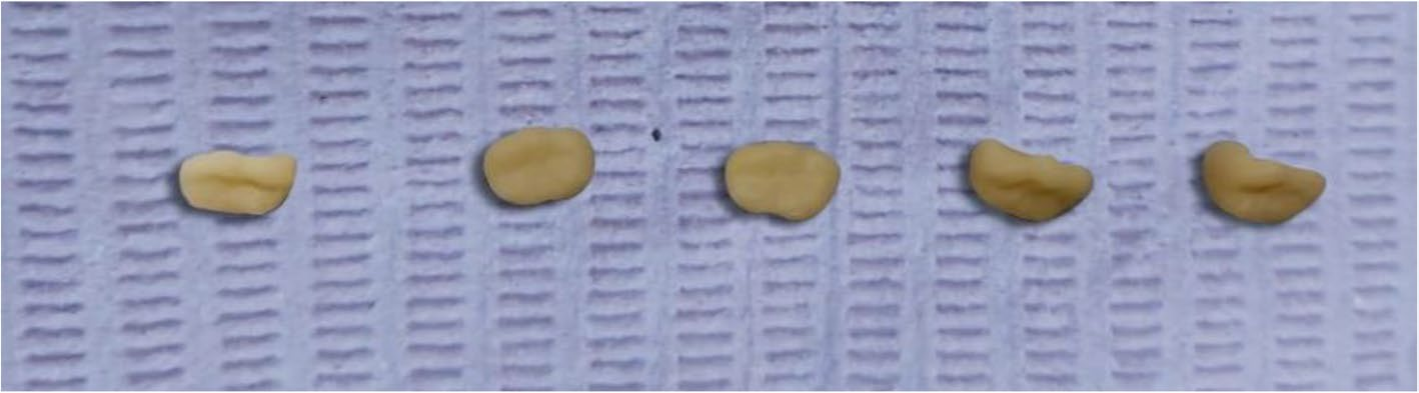
Finally sand blasted Brilliant Crios endocrown

Surface treatment via sandblasting with 50 µm Al_2_O_3_ particles under 2.5 bar pressure, at a distance 10mm, for 15 seconds was carried out. the fitting surface was ultrasonically cleaned to remove debris and dried with compressed dry air before silanization of the fitting surface (Fig. 7).

### Bonding procedure (Fig. 8a, b & c)

**Fig. 8:**
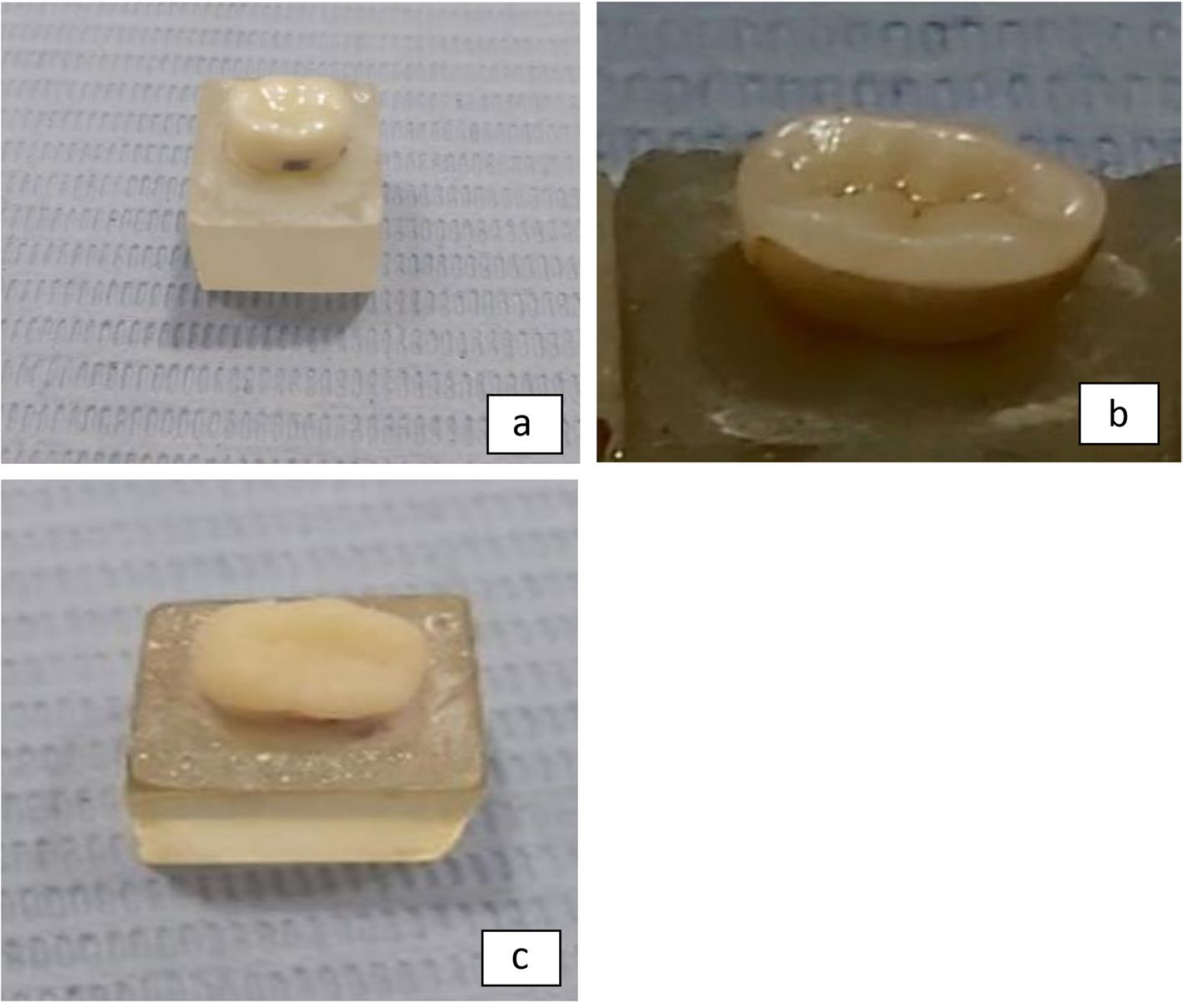
**a** Zirconia crown after bonding. **b** E-max endocrown after bonding. **c **Brilliant Crios endocrown after bonding

The adhesion technique entailed applying self-adhesive resin cement (Rely-x Unicem, 3M, Canada) to the fitting surfaces of both the restoration and the prepared dental surfaces using an automatic mixing syringe. Subsequently, the restorations were accurately positioned onto their respective prepared areas with steady finger pressure. This was followed by a quick light-curing (Woodpecker, China) step, lasting merely 2 seconds. Excess cement was meticulously removed using a scaler, and then each side of the restoration underwent a 40-second light-curing process.

### Fracture resistance assessment [23]

**Fig. 9:**
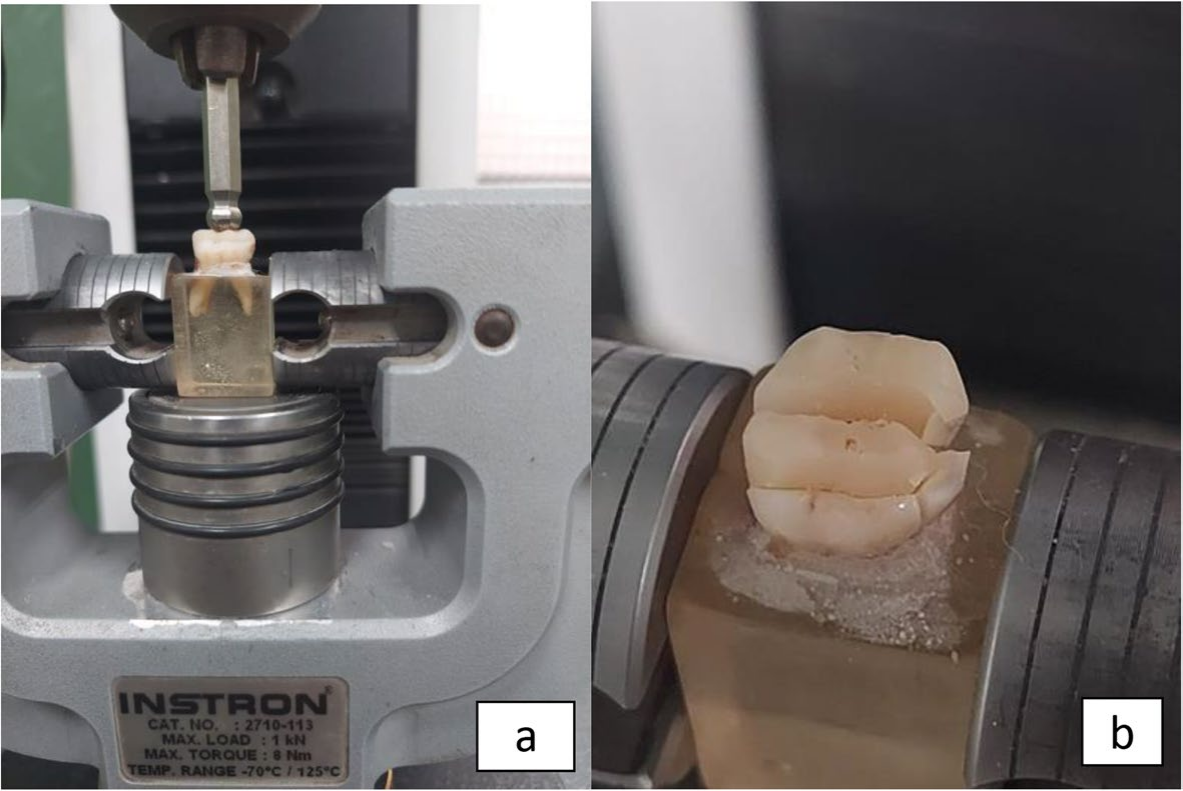
**a** & **b** Fracture resistance test by Instron machine

The center of the occlusal surface on each sample was subjected to a loading test, employing a metallic rod with a spherical tip measuring 5.8 mm in diameter. The test was conducted using a computer-controlled testing machine (Model 3345; Instron Industrial Products, Norwood, MA, US), in accordance with ISO specifications (ISO 9513), which exerted force progressively at a speed of 1 mm/min until the point of fracture. The point of fracture was determined at the first noticeable crack sound, a finding supported by a reduction observed in the load-deflection curve. This information was documented using the Bluehill Lite Software provided by Instron®, 2006-2014, US (Fig. 9a & b).

### Statistical analysis

Data compilation, verification, and organization into charts and figures were done using Microsoft Excel 2016. The dataset underwent outlier detection and normality testing to classify it as either parametric or non-parametric, using the Shapiro-Wilk and/or Kolmogorov-Smirnov tests at a 0.05 significance level (Table 1).

**Table 1:** Tests of Normality

	Kolmogorov-Smirnov^a^			Shapiro-Wilk		
	Statistic	df	Sig.	Statistic	df	Sig.
Fracture resistance	0.107	30	.200^b^	0.966	30	0.440

^a^Lilliefors Significance Correction

^b^This is a lower bound of the true significance

Descriptive statistics were presented both graphically and numerically, with parametric data represented as mean and standard deviation.

In the context of inferential statistics, a one-way Analysis of Variance (ANOVA) was executed, subsequently complemented by Duncan’s Multiple Range Test (DMRT) with a significance threshold of 0.05. The execution of these statistical analyses was facilitated using the Statistical Package for Social Sciences (SPSS), specifically IBM-SPSS version 23.0 tailored for Mac OS [24].

## Results

**Fig. 10:**
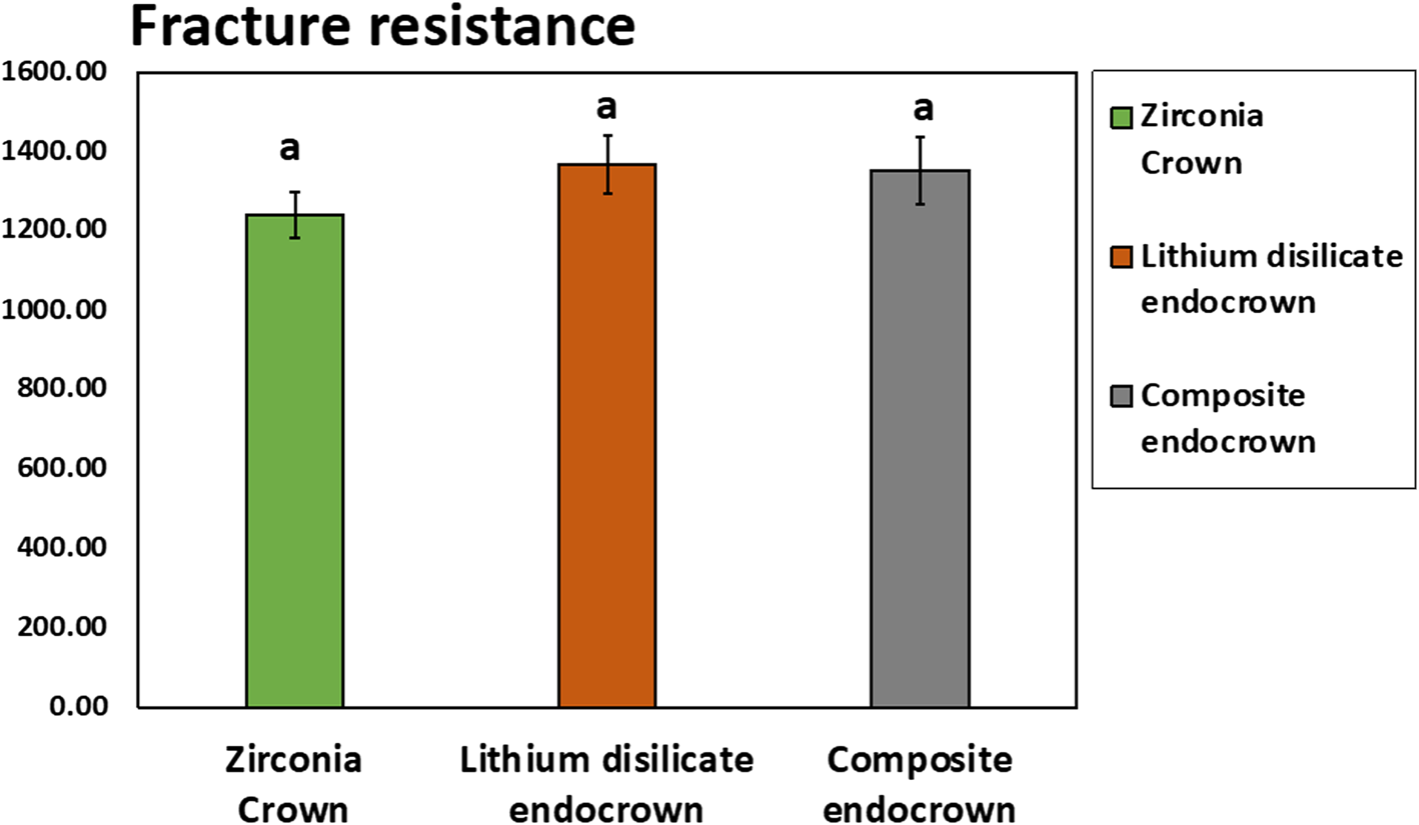
Bar chart presenting the fracture resistance of pulpotomized primary molars in Zirconia crown, Lithium disilicate endocrown, and Composite endocrown. Bars followed by similar letters aren’t significantly different according to Duncan’s Multiple Range test

According to Shapiro-Wilk Normality test (test statistic= 0.966, *p*=0.440) was non-significant i.e. data was parametric (normally distributed) and ANOVA test was applied. The comparative analysis of the three tested groups using one-way ANOVA revealed no statistically significant differences. Duncan’s Multiple Range Test indicated no significant disparities between the zirconia and E-Max groups, as well as between the zirconia and Brilliant Crios groups. Similarly, no significant difference was observed between the lithium disilicate and composite groups at the 0.05 level. The highest mean fracture resistance of pulpotomized primary molars was recorded in the lithium disilicate (E-Max) group (mean = 1367.59N), followed by the Brilliant Crios group (mean = 1349.73N), while the zirconia group exhibited the lowest mean value (mean = 1240.82N) (Table 2, Fig. 10).

**Table 2:** Descriptive statistics of the fracture resistance in the three studied materials in terms of minimum, maximum, mean, and standard deviation

**Descriptive statistic**	**Fracture Resistance (N)**		
	**Zirconia Crown **(*n*=10)	**E-Max endocrown** (*n*=10)	**Brilliant Crios endocrown **(*n*=10)
**Minimum**	919.79	1135.73	871.66
**Maximum**	1535.25	1848.10	1801.90
**Mean ±SD**	1240.82±185.34	1367.59±233.52	1349.73±267.72
**ANOVA**	0.185 ns		

- a means followed by same letter are not significantly different according to Duncan’s Multiple Range

## Discussion

Endocrowns have demonstrated their efficacy in restoring extensively damaged molars and premolars, applicable even in scenarios with considerable coronal tissue loss or the existence of occlusal risk factors, including bruxism and complex occlusal dynamics. A comprehensive review indicated that endocrowns achieve a success rate between 94% and 100%, and they possess a higher fracture strength relative to conventional restoration techniques [25].

In this study, the design of endocrowns was chosen to preserve healthy tooth structure with the help of advanced adhesive techniques. A key benefit of endocrown restorations is their typically supragingival preparation, which keeps the restoration clear of the periodontium, aiding in both periodontal health and hygiene [8, 26].

Progress in dental adhesive technology and materials, especially in the indirect composite or ceramic restorations fabricated with CAD/CAM systems, are regarded as advantageous developments in the field of pediatric dentistry. These advancements reduce time spent in the dental chair, thereby enhancing children's cooperation [4, 11].

In vitro testing was utilized to establish a controlled setting, overcoming numerous challenges often encountered in clinical testing, like the variability in human subjects. This approach yields data more representative of clinical conditions [27].

For this study, primary second molars were selected due to their significant role in mastication, occlusal development, jawbone and muscle development, and as space maintainers for permanent teeth. This factor is essential for the correct alignment of permanent teeth, influencing the overall health and well-being of children, and thus more accurately reflecting clinical situations [1].

The efficacy of the monoblock action in endocrowns relies on the availability of enamel and dentin surfaces suitable for bonding. Furthermore, the fracture resistance of these samples is greatly influenced by the surface treatment applied to both the restorations and the tooth [28].

Every tooth was individually fixed in place using self-curing acrylic resin inside a custom-made mold. The cemento-enamel junction was set 2 mm higher than, and in alignment with, the acrylic resin surface, replicating the height of normal alveolar bone to ensure stability and retention throughout the study [17].

Although primary teeth are suitable for clinical conditions, ensuring optimum standardization is difficult for zirconia group due to the variations between the hydroxyapatite structure, the size and the histology of teeth. In addition, different sizes of crowns were selected due to the use of primary teeth and a uniform amount and thickness of luting cement couldn’t be achieved [29].

In the fabrication of endocrowns, the dental specimens were shaped in compliance with the well-established clinical guidelines for all-ceramic endocrowns. This process was facilitated by the use of a specialized milling machine, ensuring uniformity in the preparation and confirming the machinability of all steps involved [2].

Research indicates that the average maximum bite force in children aged 6 to 11 years ranges from 374.4 to 433 Newtons [30]. This value is notably lower than the mean fracture resistance observed in the pulpotomized primary molars restored with the materials examined in the present study.

The enhanced fracture resistance observed in E-max endodontically treated teeth is attributed to the unique properties of interpenetrating needle-like lithium disilicate structures. These structures demonstrate superior load-bearing capacity compared to the scattered spherical constituents in resin nano ceramics (RNC) or the coalesced elements in polymer-infiltrated ceramic networks (PICN). This finding aligns with previous studies [31, 32], underscoring the superior fracture strength of lithium disilicate relative to other ceramic categories.

The extent of tooth preparation is also a critical factor in determining the fracture resistance of a restored tooth. Zirconia crowns necessitate extensive tooth preparation, in contrast to the more conservative approach required for endocrown preparation. This idea is strongly related to a study by Walczak et al. who concluded that cavity wall thickness had a significant influence on the resistance of tooth fracture [33]. Studies comparing endocrowns made of lithium disilicate reinforced ceramic with conventional crowns supported by glass fiber posts in mandibular molars have shown that endocrowns possess superior fracture strength [34, 35].

Consistent with the findings of El Makawi and Khattab (2019) [2], the current study observed no significant statistical difference between the zirconia and E-Max groups. However, they reported a higher fracture resistance in the zirconia group for pulpotomized primary molars compared to the E-max group. ElDamanhoury et al. (2015) assessed three varieties of ceramic endocrowns created using CAD/CAM technology – specifically feldspathic porcelain, E-max (lithium-disilicate), and resin nanoceramic – on upper permanent molars. The research revealed that endocrowns made of resin nanoceramic demonstrated notably greater fracture resistance and a more favorable mode of fracture, attributed to the shock-absorbing properties of the resilient resin matrix [36].

The finding of the current study was in accordance with a study by El-Mahdy et al. assessed how the crown material affects strain generation and fracture resistance of PEKK hybrid abutment crowns and they found that for the composite group, failure was observed in the crowns only in all specimens without fractures or deformations within the abutments, as the resin-based crown material acts as a shock absorber for the occlusal loads, making the strains developed in the underlying structure more bearable. while in the zirconia group, failures were more catastrophic and were within the abutments and crowns. The failure exhibited bending and deformation of the abutment and vertical fracture of the abutment and crown [37].

Cement thickness is another determinant of fracture resistance in restored teeth. Studies examining the impact of cement thickness on CAD/CAM crown failure loads found that thicker resin cement layers resulted in reduced failure loads, primarily due to polymerization shrinkage affecting crown failure resistance [34]. This is particularly relevant when considering the gap between zirconia crowns and tooth structure, where a thinner cement layer offers mechanical benefits [38].

In complex multilayered restorations, such as those involving cemented ceramics, several elements affect the mechanical stability of the combined restoration and tooth system. Key factors include the inherent strength of each component within the system (including the tooth, thickness of the luting cement layer, adhesive mechanism, and the restoration itself), the thickness of the restorative material, the comparative elasticity between the restorative material, the luting cement, and the dentin, as well as the effectiveness of the adhesive bond in terms of its strength and the potential for micro- or nano-leakage [39]. This study encounters some limitations; high cost of the materials used for endocrowns and the limited literature who investigated the use of endocrowns in primary teeth.

## Conclusion

The current study concluded that there was no significant difference in fracture resistance test of pulpotomized primary molars among the tested groups.

## Recommendations

Endocrown is a promising restoration for pulpotomized primary molars that can replace the use of zirconia crown, but further studies are needed to evaluate the physical and mechanical properties of endocrown in primary teeth, both in vitro and in vivo studies.

## Data Availability

Data cannot be shared openly but are available on request from authors:
